# The Effect of High Nicotine Dose on Maximum Anaerobic Performance and Perceived Pain in Healthy Non-Smoking Athletes: Crossover Pilot Study

**DOI:** 10.3390/ijerph20021009

**Published:** 2023-01-05

**Authors:** Peter Bartík, Peter Šagát, Jana Pyšná, Ladislav Pyšný, Jiří Suchý, Zdeněk Trubák, Dominika Petrů

**Affiliations:** 1 Health and Physical Education Department, Prince Sultan University, Riyadh 11586, Saudi Arabia; 2 Department of Physical Education and Sport, Faculty of Education, J. E. Purkyne University in Ústí nad Labem, 400 96 Ústí nad Labem, Czech Republic; 3 Department of Physical Education, Faculty of Education, Charles University, 116 39 Prague, Czech Republic

**Keywords:** anaerobic performance, high-nicotine dose, pain perception, perceived exertion, Wingate test

## Abstract

*Background:* In recent years, there has been intensive discussion about the positive effect of nicotine usage on enhancing sports performance. It is frequently applied through a non-burned tobacco form before physical activity. Nicotine is under the World Anti-Doping Agency (WADA) 2021 monitoring program. Therefore, study results that reveal either positive or negative effects are expected. This is the pilot study that reports the effect of 8 mg dose of nicotine on performance and perceived pain. *Material and Methods:* This research aimed to explore the oral intake effect of a high-nicotine dose (8 mg) on the maximum anaerobic performance and other selected physical performance parameters in healthy, well-trained adult athletes (*n* = 15, age 30.7 ± 3.6, BMI 25.3 ± 1.7). The cross-sectional study protocol included the oral administration of either sublingual nicotine or placebo tablets before the anaerobic load assessed by a standardized 30 s Wingate test of the lower limbs. Afterward, the Borg subjective perception of pain (CR 10) and Borg rating of perceived exertion (RPE) were evaluated. Wilcoxon signed-rank test was used for the analysis of data with a 0.05 level of significance. *Results:* The results revealed that oral administration of an 8 mg nicotine dose does not significantly improve any of the physical performance parameters monitored. We only reported the statistically significant positive effect in RPE (*p* = 0.03). *Conclusion:* Lower perception of pain intensity that we reported after nicotine application might be an important factor that affects performance. However, we did not report any improvement in physical performance parameters.

## 1. Background

Nowadays, any factor with an impact on maximum sports performance is crucial. One of the frequently used procedures to achieve it is an attempt to decrease perceived pain during the performance. Athletes often use analgesics, such as caffeine, amphetamine, and others, at various stages of training or competition exertion [1,2,3,4]. Substances demonstrably decreasing pain (morphine, methadone, etc.) are included in the List of Prohibited Substances [5]. However, nicotine is a much-discussed “stimulant” included in the monitoring program [6]. The alarming message is the constant increase in its use. According to the latest results, nicotine or its metabolites are detected in the urine of 23–36% of elite athletes surveyed [7]. The Marclay and Saugy study [8] confirms a 50% positive finding in elite ice hockey players. Therefore, in some sports, it exceeds the 25% incidence reported for the world population [9]. In the most recent two decades, alternative forms of nicotine have been widely used. In addition to smoking cigarettes, we also encounter smokeless tobacco (SLT) (chewing tobacco, snus, and others). This form of administration causes fast and massive absorption of the substance by the oral mucosa [10,11,12,13]. It is amply and traditionally used, especially by team athletes, players of baseball, ice hockey, football, etc., in the United States [8,14,15]. In Europe, SLT use is widespread, especially in winter sports [16,17,18]. Nicotine addiction might not be the only reason for enormous active usage. The possible positive effect on sports performance is the subject of intensive scientific debate. Nicotine is the alkaloid of simple structure, especially affecting the nervous system’s function. Several studies reported its positive effect on attention improvement, learning, and memory function [19,20,21,22,23]. Nicotine has a significant analgesic impact, as well. The outcome depends on the amount taken [24,25,26] and potentially in combination with other substances, especially alcohol [27]. Pain is a limitation factor of sports performance [28,29]. Pain tolerance might vary in beginner and professional players, the same as in athletes of contact and noncontact sports [30]. However, some other factors are important as well. The other factor might be the nicotine effect on hypothalamic function, which leads to decreased body weight [31,32]. Body composition is the limiting factor in several sports disciplines. After nicotine intake, the release of the catecholamines epinephrine and norepinephrine induces other supportive metabolic and hemodynamic effects for the realized sports performance [33]. Blood glucose level increases, and heart rate and blood pressure are boosted. The effect of nicotine on myocardial contractility in animal and human subjects was also demonstrated [34,35,36,37]. If nicotine is absorbed by smoking, the binding of carbon monoxide to hemoglobin reduces the transport capacity of the blood for oxygen [38,39,40]. However, different ways of consumption could improve the realized performance, the same as other stimulating substances, e.g., caffeine [41,42,43,44]. Thus, we hypothesize that the short-term maximal anaerobic performance observed in this study could be affected by oral nicotine application that might increase cardiac output and blood glucose levels and enhance tolerance to perceived pain during exercise.

The systematic review of physiological function changes after nicotine application revealed that the majority of 28 evaluated studies reported a significant increase in heart rate, blood pressure, and blood flow [45]. However, only one of six studies that evaluated endurance abilities reported an improvement in the monitored parameters. Several studies reported no significant ergogenic effect of lower nicotine dose (2 and 4 mg) on maximum anaerobic power by the Wingate test [46,47,48]. Conversely, the study of Johnson et al. [49] reported a positive effect after administration of 5 mg oral-dispersible nicotine strip in two repeated 30 s Wingate tests with 3 min rest between bouts. Peak and average power output were significantly greater following nicotine administration compared to placebo. Similarly, significant increases were also seen in heart rate and blood pressure following nicotine administration compared to placebo. No significant impact on pre-exercise side effect score, reaction time, rate of perceived exertion, or post-exercise blood lactate levels was observed.

Based on the above-mentioned findings, when a nicotine dose of 2–4 mg did not lead to an improvement in anaerobic performance, while intake of 5 mg increased bouts of peak and average power output during the Wingate test, we decided to assess the effect of an even higher dose (8 mg of nicotine) on maximum anaerobic performance and the subjective perception of pain and perceived exertion. Considering a high dose of nicotine administered, there has not been a similar research study conducted. This is the pilot study that reports the effect of 8 mg dose of nicotine on performance and perceived pain.

## 2. Material and Methods

The research subjects were non-smoker young and healthy men who had never developed the adherence to a smoking habit, aged 30.7 ± 3, and a BMI of 25.3 ± 4.5 in good physical condition. Participants were non-professional male athletes all recruited at the Department of Physical Education and Sport, J. E. Purkyne University in Ústí nad Labem. All are practicing regular moderate and vigorous physical activity in a range of 7–9 h a week. Detailed subject characteristics are reported in Table 1.

The inclusive criteria to keep homogeneity and the same initial parameters were the following: Participants were non-smoking athletes aged between 25–35, and their body mass index was between 20–30. All participants had a sufficient sleep (at least 7 h) before the test and performed it at the same time in the morning. All participants had a light breakfast. They signed informed consent forms and were free to step out of the research at any time. All assessments were conducted in the same laboratory environment.

All subjects underwent two assessments—the control test (CT), when the placebo was applied, and the test with the 8 mg dose of nicotine (NT). Interventions were applied in a counter-balancing manner. The chronology of the tests was assigned based on even or odd numbers. Eight participants started with nicotine application first, followed by a placebo, while seven began with a placebo application followed by nicotine administration. The period between 1st and 2nd assessment was 24 h.

An 8 mg dose of nicotine and a placebo in the form of two sublingual tablets were administered. One NiQuitin Mini tablet (GlaxoSmithKline Consumer Healthcare, Ltd., Ireland) contains 4 mg of nicotine (as a nicotine resinate). The placebo tablets were made by a pharmacist and contained the same flavor. Two nicotine or placebo tablets were taken from a prepared box and put into the mouth of the subject by a researcher. Boxes were prepared by the principal researcher and distributed to the person responsible for testing, who was not aware of the tablet’s content. The subjects for testing were unaware of which tablet they had been given and were instructed not to swallow the tablet but to let it dissolve. The tablets were applied 5 min before the start of the warm-up, followed by NT or CT.

### 2.1. Assessment Tools

Wingate anaerobic test: This is an assessment tool that measures an individual’s anaerobic capacity and anaerobic power outputs. It is the gold standard measure of anaerobic performance. The test can be conducted using only a Monark or Bodyguard cycle ergometer and a stopwatch. It requires the participant to cycle at maximal effort for 30 s.

Borg’s subjective perception of pain (CR 10): This is a general method for measuring most perceptions and experiences, including pain and perceived exertion, on a scale of 0–10.

Borg rating of perceived exertion (RPE): This is an assessment of measuring physical activity intensity level. Perceived exertion is how hard a subject feels like his body is working. It is based on the physical sensations a person experiences during physical activity, including increased heart rate, respiration or breathing rate, sweating, and muscle fatigue. Rating based on a 6 to 20 rating scale may provide a relatively good estimate of actual heart rate during physical activity.

### 2.2. Procedure

The tablets were applied 5 min prior to the beginning of the procedure (warm-up, dynamic stretching, and Wingate test) in a sitting position. Before the Wingate lower limb test, the examined subjects were warmed up on a bicycle ergometer for 5 min at the speed of 60–90 rpm with freely inserted four sprints. They were instructed on the maximum performance from the first seconds of the test and no force distribution strategy. The warm-up was followed by 3 min of dynamic stretching that targeted specific muscle groups. Afterward, participants moved to the calibrated peak bike ergometer Monark 894E. For each test, we selected a relatively high resistance of 0.104 W. kg^−1^ [50], chosen with regard to the physical performance of the subjects examined. Each tested person had undergone the Wingate test several times in the past years. The test was launched automatically after the speed of 90 rpm was reached. We created a competitive atmosphere during the trial, as maximum performance depends on motivation [51,52]. The period between both placebo and nicotine tablet administration and the beginning of the Wingate test was 20 min, considering the application 5 min prior to the procedure, 5 min warm-up, 3 min dynamic stretching, and the time needed for movement between the stations and preparations. This period corresponds to the biological onset of the nicotine administered in this form [53].

The data were evaluated using the Monark Anaerobic Test Software. The following performance parameters were assessed: peak power (P_max_ 5s) calculated by the average of the highest values at chosen 5s intervals, minimum power (P_min_ 5s) by the average of the lowest values at 5s intervals, average power (AP) and energy (E). After both anaerobic tests, we ascertained the values of CR 10 and RPE [54].

### 2.3. Statistical Analysis

Statistical analysis was performed by IBM SPSS statistical software version 25.0. Statistical analysis included a descriptive analysis of general characteristics using the mean and standard deviation (SD). The data collected were subjected to analysis by the Kolmogorov–Smirnov and Shapiro–Wilk tests to identify the distribution normality. The results show that data were not normally distributed (*p* < 0.05 for both tests). Afterward, the Wilcoxon signed-rank test with median values was performed with a 0.05 level of significance.

## 3. Results

In Table 2, we provide the control test (CT) results when placebo tablets were administered and the test after nicotine absorption (NT). The parameters of muscle work are expressed in watts per kilogram of weight (W.kg^−1^), particularly peak power (maximum power, P_max_), average power (AP), the average value from the best 5 s (P_max_ 5s), and the average value from the worst 5 s (P_min_ 5s). The total energy exerted is represented by E in kilojoules. The values of CR-10 and RPE are reported afterward.

The results of the Wilcoxon test, calculated according to the median value, are reported in Table 3. In the case of performance comparison, we did not identify any statistical significance when comparing CT and NT groups. We only reported a statistically significant difference in the rating of perceived exertion (*p* = 0.031). The perceived pain intensity was lower in NT.

## 4. Discussion

The study results revealed that oral administration of an 8 mg high-nicotine dose does not significantly improve physical performance parameters monitored by the Wingate test. We only reported a statistically significant difference in the rating of perceived exertion (*p* = 0.031). The perceived pain intensity was lower in NT. Nicotine is included in the WADA monitoring program because of its possible performance-increasing effect [6]. If this effect is scientifically proven, we can expect its inclusion in the list of forbidden substances. The positive effect of nicotine intake on athletic performance can be predicted. This depends on the type of physical activity, the amount of nicotine, the product type, and the application method [45]. Our outcomes have not demonstrated a statistically significant increase in sports performance as a result of administering a dispersible tablet with a high dose of 8 mg of nicotine. Therefore, the outcome of this study corresponds with the results of other studies, monitoring the effect of lower doses of nicotine (2–4 mg) on maximal anaerobic performance [46,47,48]. Furthermore, we did not report any possible beneficial effect with a higher nicotine dose such as in the study with improved physical performance when 5 mg of nicotine was applied [49]. A possible explanation for this fact might be the difference in assessment protocol. While in the above-mentioned study, the two repeated 30 s Wingate tests with 3 min rest between bouts were used for assessment, we applied a single 30 s Wingate test. In the first case, the testing period was longer with higher difficulty, so the nicotine effect might present itself with higher significance. The other possible explanation for different results might be the way of nicotine application. While in the above-mentioned study, the oral strips were applied as a source of nicotine, in our study, we used nicotine tablets.

One of the reasons why athletes smoke, chew tobacco, or otherwise consume nicotine might be its effect on aerobic and partly also anaerobic metabolism. Nicotine increases cardiac output, muscle blood flow, and the availability of glucose [34,35,36,37]. The other significant effect of nicotine that might enhance sports performance is on cognitive functions [19,20,21,22]. However, nicotine’s beneficial cognitive effects have implications for smoking initiation and maintenance of tobacco dependence [19].

It is important to note that this amount of nicotine has a statistically significant positive effect on RPE in our study. It can be assumed that this substance may affect some central nervous system functions. As confirmed by some studies [24,25,26], this certainly leads to a higher tolerance for pain. At the same time, the realization of maximum anaerobic performance is often limited by this kind of perception. There is still a lack of studies devoted to this topic. The fact that our single 30 s Wingate test reported a null result in a physical performance boost, but it does in RPE decrease, might be affected by the type of assessment. It is possible that by modification of the assessment length and load, we would report different results. This should be a subject and focus of future research.

Nicotine activates brain systems underlying reward and antinociception but simultaneously elicits aversive sensory effects, including oral irritation and pain, bitter taste, and other unpleasant side effects mediated largely by nicotinic acetylcholine receptors (nAChRs) [55]. Nicotine potently decreases allodynia, which could also lead to increased nicotine consumption in chronic pain subjects [56]. However, it must be emphasized that nicotine can negatively impact human health, e.g., by substantial hemodynamic changes [33]. Cigarette smoking has been identified as a risk factor in the onset of back pain, sciatica, rheumatoid arthritis headache, oral pain, and diabetic peripheral neuropathy [57,58,59,60,61,62]. Moreover, lifetime cigarette exposure has been positively associated with the risk of developing persistent pain, pain severity, and experimental pain reactivity [63,64]. Therefore, nicotine acts as an analgesic when used acutely or short-term, while long-term exposure or nicotine withdrawal (similar to smoking cessation) results in hyperalgesia [64]. Together with maximum exertion, the response of the cardiovascular system is stronger. Our experience is the following: After exertion with the intervention, subjects have, in several instances, reported reactions such as a sense of pressure in the head area or nausea. Therefore, we assume that a single higher dose (8 mg) in individuals not using nicotine does not have a favorable effect on the cardiovascular and digestive systems.

With regard to the data reported and also the generally reported negative health consequences, we cannot recommend nicotine for the purpose of increasing anaerobic sports performance. However, the intake of this amount of nicotine also had a significant positive effect on the Borg’s rating of perceived exertion. Some studies have already demonstrated nicotine’s positive effect on reduced fatigue perception and higher pain tolerance [65,66]. The suppression of perceived pain can be a limiting factor in realizing maximum performance, and it is necessary to investigate this possible effect further.

This research has its limitation as well. It is acknowledged that a sample size of *n* = 15 is relatively small. However, precise and directional statistical results were observed even with this sample. Furthermore, the homogeneity of this group is granted due to specific inclusion criteria.

## 5. Conclusions

The lower perception of pain intensity that we reported after the 8 mg nicotine dose application might be an important factor that affects performance. However, we did not report any improvement in physical performance parameters.

## Figures and Tables

**Table 1:** Subject’s characteristics (mean ± standard deviation).

Number of Subjects	Age	Height	Weight	BMI
15	30.7 ± 3	183.3 ± 5.5 cm	85.1 ± 10.8 kg	25.3 ± 4.5

BMI—body mass index.

**Table 2:** Wingate test and perceived pain results (mean ± SD).

	P_max_ [W.kg^−1^]	AP [W.kg^−1^]	P_max_ 5s [W.kg^−1^]	P_min_ 5s [W.kg^−1^]	E [kJ]	CR-10	RPE
**CT**	19.10 ± 2.35	12.23 ± 1.34	16.19 ± 1.86	8.62 ± 1.05	48.16 ± 8.23	7.93 ± 2.55	19.07 ± 1.79
**NT**	18.97 ± 2.49	12.18 ± 1.40	15.81 ± 1.92	8.66 ± 1.10	45.73 ± 9.71	7.53 ± 2.42	17.40 ± 3.76

Note: **CT** = Control test with placebo; **NT** = Test with dose of 8 mg nicotine, **P_max_** [W.kg^−1^] = Peak power, maximum power, **AP** [W.kg^−1^] = Average power, **P_max_ 5s** [W.kg^−1^] = Maximum power, average from the best 5s; **P_min_ 5s** [W.kg^−1^] = Minimum power, average from the worst 5s, **E** [kJ] = Energy expenditure during the Wingate test, **CR-10** = Subjective perception of pain, **RPE** = Borg rating of perceived exertion.

**Table 3:** Wilcoxon signed-rank test results.

	P_max_ [W.kg^−1^]	AP [W.kg^−1^]	P_max_ 5s [W.kg^−1^]	P_min_ 5s [W.kg^−1^]	E [kJ]	CR-10	RPE
**CT**	19.08	12.27	16.05	8.95	50.79	10.00	20.00
**NT**	18.48	12.09	15.48	8.65	42.24	7.00	20.00
z	0.682	0.341	1.676	–0.710	1.022	1.057	2.205
*p*-value	0.524	0.762	0.097	0.498	0.330	0.375	0.031 *
r	0.124	0.062	0.306	0.130	0.187	0.193	0.403

Note: **CT** = Control test; **NT** = Test with dose of 8 mg nicotine, **P_max_** [W.kg^−1^] = Peak power, maximum power, **AP** [W.kg^−1^] = Average power, **P_max_ 5s** [W.kg^−1^] = Maximum power, average from the best 5s; **P_min_ 5s** [W.kg^−1^] = Minimum power, average from the worst 5s, **E** [kJ] = Energy expenditure during the Wingate test, **CR-10** = Subjective perception of pain, **RPE** = Borg rating of perceived exertion, z = Standard error of the mean, r = Effect size. * We only reported a statistically significant difference in the rating of perceived exertion (*p* = 0.031).

## Data Availability

The data presented in this study are available on request from the corresponding author.
